# Serum Amyloid A and Clusterin as Potential Predictive Biomarkers for Severe Hand, Foot and Mouth Disease by 2D-DIGE Proteomics Analysis

**DOI:** 10.1371/journal.pone.0108816

**Published:** 2014-09-30

**Authors:** Jianjun Liu, Peiwu Huang, Yaqing He, Wen-Xu Hong, Xiaohu Ren, Xifei Yang, Yanxia He, Wenjian Wang, Renli Zhang, Hong Yang, Zhiguang Zhao, Haiyan Huang, Long Chen, Dejian Zhao, Huixia Xian, Fang Yang, Dongli Ma, Linqing Yang, Yundong Yin, Li Zhou, Xiaozhen Chen, Jinquan Cheng

**Affiliations:** 1 Shenzhen Center for Disease Control and Prevention, Shenzhen, China; 2 Shenzhen Children’s Hospital, Shenzhen, China; Medical Sciences Campus, Puerto Rico

## Abstract

Hand, foot, and mouth disease (HFMD) affects more than one million children, is responsible for several hundred child deaths every year in China and is the cause of widespread concerns in society. Only a small fraction of HFMD cases will develop further into severe HFMD with neurologic complications. A timely and accurate diagnosis of severe HFMD is essential for assessing the risk of progression and planning the appropriate treatment. Human serum can reflect the physiological or pathological states, which is expected to be an excellent source of disease-specific biomarkers. In the present study, a comparative serological proteome analysis between severe HFMD patients and healthy controls was performed via a two-dimensional difference gel electrophoresis (2D-DIGE) and matrix-assisted laser desorption/ionization time-of-flight mass spectrometry (MALDI-TOF MS) strategy. Fifteen proteins were identified as differentially expressed in the sera of the severe HFMD patients compared with the controls. The identified proteins were classified into different groups according to their molecular functions, biological processes, protein classes and physiological pathways by bioinformatics analysis. The up-regulations of two identified proteins, serum amyloid A (SAA) and clusterin (CLU), were confirmed in the sera of the HFMD patients by ELISA assay. This study not only increases our background knowledge about and scientific insight into the mechanisms of HFMD, but also reveals novel potential biomarkers for the clinical diagnosis of severe HFMD.

## Introduction

Hand foot and mouth disease (HFMD) was first reported in New Zealand in 1957 and is an unremarkable illness that commonly occurs in young children, primarily those who are under the age of 10 years [1]. Two closely related viruses, coxsackievirus A16 (CVA16) and enterovirus 71 (EV71), are identified as the most frequent pathogens of HFMD [2]. Epidemiological data have shown that the incidence of HFMD had increased in Southeast Asia in the past 20 years. A well- known and large outbreak of HFMD occurred in Taiwan in 1998 [3]. In the most common manifestation of HFMD, children typically present with vesicular exanthema on the feet, hands and mouths, that leads to discomfort and feeding difficulties. Severe health consequences or death may occur in severe HFMD cases, owing to complications such as encephalitis, aseptic meningitis, acute flaccid paralysis, myocarditis, autonomic nervous system dysregulation and pulmonary edema or hemorrhage [4], [5].

In China, the numbers of HFMD cases and fatal HFMD cases has increased every year, and multiple major HFMD outbreaks have occurred in several provinces [6]. HFMD affects more than one million children and is responsible for several hundred child deaths every year, which has caused widespread concerns in society. Since severe HFMD cases can cause neurologic diseases and significant fatalities, there is an urgent need for additional markers to improve the diagnostic accuracy of severe HFMD and provide timely treatment [6].

Serum is expected to be an excellent source of protein biomarkers because it circulates through, or comes into contact with all tissues. During such contact, the serum is likely to pick up proteins secreted or shed by tissues [7]. Human serum can reflect physiological or pathological states and provide robust correlation in the entire body [8]. Peptidome analyses based on mass spectrometric screening methods offered a high-throughput approach for the discovery of new potential biomarkers, which can be useful in the clinical diagnosis and disease treatment [9], [10]. Many researchers have attempted to provide disease-specific biomarkers for the early detection of diseases, drug susceptibilities, and the evaluation of prognoses [11]–[13]. Proteomics-based techniques, such as surface-enhanced laser desorption/ionization (SELDI) and high-performance liquid chromatography (HPLC), have been used to study serum biomarkers in various diseases [14]. Two-dimensional (2D) gel electrophoresis is a powerful technique that enables the simultaneous visualization of relatively large portions of the proteome. By the internal standards and fluorescence labeling, two-dimensional difference gel electrophoresis (2D-DIGE) has the advantages of adequate sensitivity, high reproducibility and minimized experimental variation over traditional 2-DE proteomics [15].

In the present study, a comparative serological proteome analytic comparison of severe HFMD patients and healthy controls was performed via a 2D-DIGE and matrix-assisted laser desorption/ionization time-of-flight mass spectrometry (MALDI-TOF MS) strategy. Fifteen proteins were identified as differentially expressed in the sera of severe HFMD patients compared with controls. The classifications of the identified proteins were analyzed with PANTHER (Protein Analysis through Evolutionary Relationships) [16], and the biological pathways were analyzed with DAVID (Database for Annotation, Visualization and Integrated Discovery) [17], [18]. The up-regulations of two identified proteins, serum amyloid A (SAA) and clusterin (CLU), were confirmed in the serum of HFMD patients by an ELISA assay. This study not only increases our background knowledge about and scientific insight into the mechanisms of HFMD but also reveal novel potential biomarkers for the clinical diagnosis of severe HFMD.

## Materials and Methods

### Ethics Statement

The study protocol was conducted in accordance with the declaration of Helsinki [19] and was approved by the Medical Ethics Committee of Shenzhen Center for Disease Control and Prevention (SZCDC), Shenzhen, P. R. China. Written informed consent was obtained from all parents, caretakers or guardians on behalf of the minors/children enrolled in this study.

### Reagents, instruments and software

Research-grade acetonitrile (ACN), trifluoroacetic acid (TFA), ammonium bicarbonate, ammonium acetate, acetone, ethanol, and methanol were purchased from Sigma-Aldrich (St. Louis, Missouri, USA). The 2D Quant Kit was purchased from GE Healthcare (Piscataway, New Jersey, USA). Sequencing Grade Modified Trypsin was purchased from Promega (Madison, Wisconsin, USA). The UltrafleXtreme MALDI-TOF/TOF mass spectrometer and MTP 384 target plate polished steel were acquired from Bruker Daltonics Inc (Billerica, Massachusetts, USA). The following software programs were used: FlexControl Version 3.3, FlexAnalysis Version 3.3, BioTools Version 3.2 (Bruker Daltonics Inc, Billerica, Massachusetts, USA).

### Study subjects

All the sera from severe HFMD patients, as classified according to the diagnostic criteria for the “hand, food and mouth disease prevention control guide (2010 Edition) ” issued by the Ministry of Health, China (http://www.moh.gov.cn/yzygj/s3593g/201306/6d935c0f43cd4a1fb46f8f71acf8e245.shtml), were collected from April 2012 to September 2012 at the Shenzhen Children’s Hospital and Shenzhen BaoAn People Hospital. Twenty patients (13 males and 7 females; 2.01±0.84 years of age (mean ± standard deviation)) were hospitalized for treatment (Table 1 and Table S1). Samples from all patients underwent the ELISA assay and the samples from 4 males and 4 females (1.74±0.44 years of age) were underwent the 2D-DIGE analysis. Twenty age-matched (±2 years) normal subjects (13 males and 7 females; 3.51±0.69 years of age) were selected. Similarly, ELISA was performed for all normal subjects and 8 subjects (4 males and 4 females, 3.28±0.40 years of age) were assigned to the 2D-DIGE set.

**Table 1:** The clinical characteristics of the severe HFMD and normal groups.

Groups	Items	2D-DIGE set	ELISA set
Severe HFMD	number	8	20
	male/female	4/4	13/7
	average	1.74±0.44	2.01±0.84
Normal	number	8	20
	male/female	4/4	13/7
	average age	3.28±0.40	3.51±0.69

### Sample collection

Fasting blood samples (2 mL) were collected into anticoagulant-free tubes. The samples were coded and transported on ice to the laboratory. The tubes were centrifuged at 2500 rpm at 20°C for 10 min within a 30-min time frame. Serum from each patient sample was then collected, aliquoted and stored at –80°C until the time of analysis. Each serum sample underwent no more than 3 freeze/thaw cycles prior to analysis. For the 2D-DIGE analysis, 8 patients and 8 control samples were included. Equal volumes of sera from two individual samples of the same group were pooled as one biological replicate. Four biological replicates were generated for each group. The levels of SAA and CLU from 20 patients and 20 control samples were confirmed in ELISA assay.

### Sample Preparation for 2-D Electrophoresis

Serum samples were processed using the ProteoExtract Albumin/IgG removal kit (EMD Biosciences, Billerica, Massachusetts, USA) according to the manufacturer’s instructions. In brief, 40 µL of each pooled serum sample was diluted 10-fold with binding buffer, loaded to the resin column and allowed to pass by gravity-flow. The column was washed twice with 600 µL of binding buffer. The combined flow-through fraction contained the albumin/IgG-depleted sample. Approximately above 90% of both the albumin and IgG is removed by this method. Each depleted serum fraction was concentrated and desalted by ultrafiltration using a 3 kDa cut off Centrifugal Filter Device (Millipore, Billerica, Massachusetts, USA). The final serum sample protein concentrations were determined with the 2-D Quant kit (GE Healthcare, Piscataway, New Jersey, USA), which is designed for the accurate determination of protein concentration in samples prepared for electrophoresis techniques. The depleted sera were collected and stored at −80°C until analysis.

### Reconstitution of CyDyes and Labeling of Protein

Protein extracts were labeled with three CyDye DIGE fluors, Cy2, Cy3 and Cy5, for 2D-DIGE technology according to the manufacturer’s recommended protocols. Briefly, lyophilized CyDyes (GE Healthcare, Piscataway, New Jersey, USA) were resuspended in 5 µL of anhydrous N,N-dimethylformamide (Sigma-Aldrich, St. Louis, Missouri, USA) to produce a stock solution of 1 mM. A working solution of 0.20 mM was generated by diluting the stock solution with DMF. A total of 25 µg of protein was labeled with 200 pmol of with Cy3 or Cy5 using the minimal labeling technique. To avoid bias due to different fluorescent characteristics at different wavelengths, half of the samples from each group were labeled by Cy3 and Cy5, respectively. An internal standard was generated by pooling equal amounts of all of the samples to control for gel-to-gel variation and was labeled with Cy2. The labeling reaction was conducted on ice in darkness for 30 min and then quenched with 1 µL of 10 mM lysine (Sigma-Aldrich, St. Louis, Missouri, USA) for 10 min on ice in the dark. The Cy3- and Cy5-labeled samples were mixed with 25 µg of the Cy2-labelled internal standard and resolved in one gel. Equal volumes of 2 × sample buffer were added to each pooled sample (7 M urea, 2M thiourea, 4% w/v CHAPS, 2% w/v DTT, 2% v/v IPG buffer pH 4–7) and left on ice for 10 min prior to bringing the total volume of the sample to 450 µL with rehydration buffer (8 M urea, 2% w/v CHAPS, 0.28% w/v DTT, 0.5% v/v IPG buffer pH 4–7, 0.002% w/v bromophenol blue).

### 2-D Electrophoresis

For 2-D electrophoresis, immobilized pH gradient (IPG) strips (24 cm, pH 4–7) (GE Healthcare, Piscataway, New Jersey, USA) were rehydrated in the sample buffer at 30 V for 12 h using an Ettan IPGphor 3 isoelectric focusing system (GE Healthcare, Piscataway, New Jersey, USA). Later, the samples were focused at 500 V for 1 h, 1000 V for 1 h, and 8000 V Grd for 5 h, 8000 Stp until a total of 80 000 VhT at 20°C. Following isoelectric focusing, each strip was equilibrated for 15 min at room temperature in equilibration buffer (6 M urea, 75 mM Tris-HCl pH 8.8, 29.3% v/v glycerol, 2% w/v SDS, 0.002% w/v bromophenol blue) containing 1% w/v DTT and then equilibrated for another 15 min in equilibration buffer containing 2.5% w/v iodoacetamide. The equilibrated strips were then embedded in agarose sealing solution (25 mM Tris, 192 mM glycine, 0.1% SDS, 0.5% w/v low melting point agarose, 0.002% bromophenol blue) on top of 12.5% SDS-PAGE gels which were prepared in on low fluorescence borosilicate glass plate (GE Healthcare, Piscataway, New Jersey, USA). The second dimension separation was conducted on an Ettan DALT six electrophoresis system (GE Healthcare, Piscataway, New Jersey, USA) at 15°C with the program of 1 W/gel for 1 h, followed by 11 W/gel for 6 h. For spots picking and *in-gel digestion*, a IPG strips (24 cm, pH 4–7) loading of 1000 mg of unlabeled proteins was performed in parallel and stained with Coomassie brilliant blue G-250 (Bio-Rad, Hercules, California, USA).

**Table 2:** Differentially expressed proteins identified by mass spectrometry (MS) after 2-D DIGE analysis.

Entry	Master No.	Protein ID	Protein Name	*t*-test	Av. Ratio	Score	Coverage	Mass	pI	Sequence identified
**Up-regulated proteins in severe HFMD groups**										
1	1357	SAA_HUMAN	Serum amyloid A protein	0.018	45.4	333	27	13581	6.4	EANYIGSDKYFHAR AYSDMREANYIGSDKYFHAR
	1359	SAA_HUMAN	Serum amyloid A protein	0.0085	31.19	316	39	13581	6.4	RGPGGAWAAEVISDAR EANYIGSDKYFHAR FFGHGAEDSLADQAANEWGR
2	928	CLUS_HUMAN	Clusterin	0.00029	6.13	322	3	188569	6	EGDDDRTVCR ELDESLQVAER EILSVDCSTNNPSQAK
3	1042	HPT_HUMAN	Haptoglobin	0.0029	2.33	337	15	45861	6.1	VTSIQDWVQK HYEGSTVPEKK NANFKFTDHLK YVMLPVADQDQCIR
	1007	HPT_HUMAN	Haptoglobin	0.0028	2.16	457	16	45861	6.1	YVMLPVADQDQCIR VVLHPNYSQVDIGLIK
	987	HPT_HUMAN	Haptoglobin	0.025	1.72	496	17	45861	6.1	SCAVAEYGVYVK YVMLPVADQDQCIR
	937	HPT_HUMAN	Haptoglobin	0.013	1.62	359	12	45861	6.1	YVMLPVADQDQCIR VMPICLPSKDYAEVGR SPVGVQPILNEHTFCAGMSK
	1237	HPT_HUMAN	Haptoglobin	0.047	1.56	187	14	45861	6.1	LRTEGDGVYTLNDK AVGDKLPECEAVCGKPK TEGDGVYTLNNEKQWINK
4	832	A2GL_HUMAN	Leucine-richalpha-2-glycoprotein	0.015	1.96	565	22	38382	6.5	ALGHLDLSGNR DLLLPQPDLR
**Down-regulated proteins in severe HFMD groups**										
5	1131	APOA1_HUMAN	Apolipoprotein A-I	9.70E-05	−8.13	307	18	30759	5.5	WQEEMELYR VQPYLDDFQKK
	1163	APOA1_HUMAN	Apolipoprotein	0.0092	−2.4	273	20	30759	5.5	DEPPQSPWDR
			A-I							WQEEMELYR LLDNWDSVTSTFSK
	1143	APOA1_HUMAN	Apolipoprotein	0.0014	−2.22	378	20	30759	5.5	DEPPQSPWDR
			A-I							WQEEMELYR LLDNWDSVTSTFSK
	1142	APOA1_HUMAN	Apolipoprotein	0.007	−2.21	373	20	30759	5.5	DEPPQSPWDR
			A-I							WQEEMELYR DSGRDYVSQFEGSALGK
	1139	APOA1_HUMAN	Apolipoprotein	0.0022	−1.78	256	17	30759	5.5	DEPPQSPWDR
			A-I							WQEEMELYR LLDNWDSVTSTFSK
6	407	A2MG_HUMAN	Alpha-2-macroglobulin	0.0075	−2.84	371	5	164614	6	QGIPFFGQVR VGFYESDVMGRGVPIPNKVIFIR LVHVEEPHTETVR
	409	A2MG_HUMAN	Alpha-2-macroglobulin	0.0097	−2.82	380	4	164614	6	QGIPFFGQVR VGFYESDVMGRGVPIPNKVIFIR LVHVEEPHTETVR
	413	A2MG_HUMAN	Alpha-2-macroglobulin	0.022	−2.56	455	6	164614	6	QGIPFFGQVR VGFYESDVMGRVTAAPQSVCALR SGGRTEHPFTVEEFVLPK
	406	A2MG_HUMAN	Alpha-2-macroglobulin	0.037	−2.32	435	5	164614	6	QGIPFFGQVR VGFYESDVMGRGVPIPNKVIFIR LVHVEEPHTETVR
7	846	FIBG_HUMAN	Fibrinogen	0.0056	−2.62	336	11	52106	5.3	VGPEADKYR
			gamma chain							VELEDWNGR
										DNCCILDER YLQEIYNSNNQK
	845	FIBG_HUMAN	Fibrinogen	0.011	−2.37	370	15	52106	5.3	VGPEADKYR
			gamma chain							VELEDWNGR
	859	FIBG_HUMAN	Fibrinogen	0.0024	−2.16	175	6	52106	5.3	VGPEADKYR
			gamma chain							DNCCILDER YLQEIYNSNNQK
8	1348	APOC3_HUMAN	Apolipoprotein C-III	0.0066	−2.57	270	19	10846	5.1	DALSSVQESQVAQQAR SEAEDASLLSFMQGYMK TAKDALSSVQESQVAQQAR
	1360	APOC3_HUMAN	Apolipoprotein C-III	0.024	−1.98	157	19	10846	5.1	DALSSVQESQVAQQAR TAKDALSSVQESQVAQQAR
9	689	ANT3_HUMAN	Antithrombin-	0.00082	−2.3	290	10	53025	6.3	ANRPFLVFIR
			III							EVPLNTIIFMGR DIPMNPMCIYR
10	1299	TTHY_HUMAN	Transthyretin	0.0045	−1.99	382	25	15991	5.4	AADDTWEPFASGK KAADDTWEPFASGK GPTGTGESKCPLMVK
11	480	A2AP_HUMAN	Alpha-2-antiplasmin	0.0058	−1.89	160	9	54873	5.9	LCQDLGPGAFR LVPPMEEDYPQFGSPK
12	554	ALBU_HUMAN	Serum albumin	0.011	−1.89	220	9	71317	5.9	AVMDDFAAFVEK RHPDYSVVLLLR RPCFSALEVDETYVPK
13	1404	APOA2_HUMAN	Apolipoprotein	0.045	−1.78	126	21	11282	7.1	SKEQLTPLIK
			A-II							VKSPELQAEAK
										SKEQLTPLIK
14	579	KNG1_HUMAN	Kininogen-1	0.0036	−1.66	287	6	72996	6.4	QVVAGLNFR
										YFIDFVAR
										RPPGFSPFR YNSQNQSNNQFVLYR
	612	KNG1_HUMAN	Kininogen-1	2.60E-	−1.54	350	6	72996	6.4	QVVAGLNFR
				05						YFIDFVAR
										RPPGFSPFR KYNSQNQSNNQFVLYR
15	529	A1BG_HUMAN	Alpha-1B-glycoprotein	0.022	−1.51	259	11	54809	5.5	HQFLLTGDTQGR CEGPIPDVTFELLR LELHVDGPPPRPQLR

### Image Analysis

After the 2-D elelctrophoresis was completed, the gels were immediately scanned using a Typhoon Trio Variable Mode Imager (GE Healthcare, Piscataway, New Jersey, USA). Briefly, the Cy2, Cy3 and Cy5-labelled images for each gel were scanned with excitation/emission wavelengths of 488/520, 532/580 and 633/670 nm, respectively. All 12 protein spot maps from the four gels were loaded into the DeCyder 2D 6.5 software (GE Healthcare, Piscataway, New Jersey, USA) for image analysis. The threshold for spot detection was set at 1000 to estimate the number of protein spots present in the different gels using the differential in-gel analysis (DIA) module, and the spot volumes of the Cy2 map were used to normalize the corresponding spot volume of Cy3 and Cy5 maps from the same gel. Manual spot matching between the different gels was carried out using the four internal standard spot maps before automatic matching of all of the spot maps with the biological variation analysis (BVA) module. After matching, the normalized volumes of the spots were calculated and compared between the severe HFMD patients and the controls. Protein spots with significant differences in abundance (more than 1.5-fold) were selected from the stained gels for spot picking.

### Spot picking and in-gel digestion

The protein spots exhibiting significant changes (*p*<0.05) in expression between the severe HFMD samples and the control samples were manually excised from the Coomassie brilliant blue- stained gel and destained with 50% acetonitrile in 25 mM ammonium bicarbonate followed by dehydration in 100% acetonitrile. After the reagents were removed, the gel pieces were digested with 0.15 µg of sequencing-grade trypsin (Promega, Madison, Wisconsin, USA) in 15 µL digestion buffer containing 25 mM ammonium bicarbonate. The mixture was incubated overnight at 37°C and stored at −80°C prior to protein identification.

### Mass Spectrometry

Protein identification was carried out on an UltrafleXtreme MALDI-TOF/TOF Mass Spectrometer (Bruker Daltonics Inc, Billerica, Massachusetts, USA). Briefly, 2 µL of peptide mixture was dripped on a polished steel plate, air dried and covered with 1 µL of matrix HCCA (10 mg/mL in 70% CH3CN/0.1% TFA) before being sent to the machine. Standard peptide mixtures (Bruker Daltonics Inc, Billerica, Massachusetts, USA) were served as internal standard calibration. Both the peptide mass fingerprinting (PMF) and fragment spectrums of the parent ions (MS/MS) were obtained by the FlexControl software and processed by the FlexAnalysis before being sent to MASCOT search engine of BioTools (Bruker Daltonics Inc, Billerica, Massachusetts, USA). The peptide mass fingerprint combined MS/MS spectra were searched against the SwissProt database and MASCOT Version 2.2 (Matrix Science, Boston, Massachusetts, USA) with the following parameters: Homo sapiens, trypsin cleavage, two missed cleavages were allowed, carbamidomethylation as a fixed modification, oxidation of methionines was allowed as a variable medication, peptide mass tolerance of 100 ppm, and a fragment tolerance of 0.5 Da. Proteins matching more than 2 peptides and with a MASCOT score higher than 63 were considered statistically significant (*p*<0.05).

### Bioinformatics analysis

Protein ontology classification was performed by importing the proteins into the protein analysis through evolutionary relationships (PANTHER) classification system (http://www.pantherdb.org/, SRI International, Menlo Park, California, USA) [16]. The proteins were grouped according to their molecular functions, biological processes and protein classes.

Pathway analysis of the identified proteins was performed using the database for annotation, visualization and integrated discovery (DAVID), version 6.7 (http://david.abcc.ncifcrf.gov/home.hsp) [17], [18] to better understand of the participation of the altered proteins in various physiological pathways.

### Enzyme-linked immunosorbent assay (ELISA)

To confirm the differential expression of the identified proteins, a ELISA-based validation experiments were performed using commercially available ELISA Kit for human serum amyloid A (Abcam, Cambridge, UK) and human clusterin (Uscn Life Science, Wuhan, China) according to the manufacturer’s specifications. Briefly, the crude sera from 20 severe HFMD patients and controls were included in the ELISA assays. Each sample was assayed in duplicate. The serum protein concentrations were calculated from kit-specific standard curves generated by CurveExpert 1.4 software, and the assay results were analyzed using the GraphPad Prism software and SPSS v10 software package (SPSS Inc., Chicago, Illinois, USA).

### Statistical analysis

The data are expressed as the mean ± the SD and were analyzed using SPSS statistical software (SPSS Inc., Chicago, Illinois, USA). Student’s *t*-tests were used to compare pairs of means. ANOVA followed by Student Newman-Keuls post-hoc tests were used to compare three or more means. The level of significance was set as *p*<0.05.

## Results

### 2-D DIGE analysis and identification of differential protein expression

**Figure 1:**
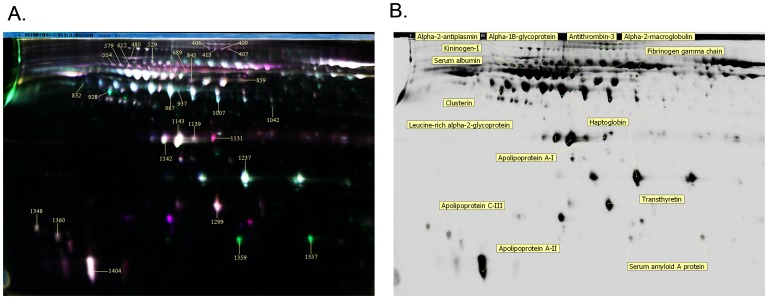
2D-DIGE analysis of the protein spots were differentially expressed between the severe HFMD groups and the healthy controls. (A) The proteins extracted from the HFMD and control samples were labelled with Cy3 and Cy5, respectively. An internal standard protein sample (a mixture of severe HFMD and control samples) was labeled with the Cy2 dye. The green spots represent up-regulated proteins, while the red spots represent down-regulated proteins in the severe HFMD samples compared with the control samples. The yellow arrow represents the identified spots that showed significantly different expression between the severe HFMD and control samples. The number in the figure corresponds to the master number presented in Tables 2. (B) Differentially expressed proteins identified in 2-DE gels.

Serum albumin and immunoglobulin G are the most abundant proteins in plasma, which are able to mask the low-abundant proteins and limit the amount of proteins that can be identified by 2D-DIGE analysis. To increase the sensitivity of the visualization of low abundant proteins, albumin and IgG were specifically depleted using the ProteoExtract Albumin/IgG removal kit. A characterization of the severe HFMD patients’ samples from which sera were obtained for DIGE analysis is presented in Fig. 1A. After the 2-D DIGE analysis, differential protein spots were detected using the DeCyder analysis software. Protein spots with at least 1.5-fold differential expression were selected for MS identification.

A total of 15 proteins (4 up-regulated and 11 down-regulated) were identified as differentially expressed between the severe HFMD samples and the controls (Table 1 and Fig. 1B). The selected tandem mass spectra of the peptides originating from the SAA and CLU are shown in Figure S1 and S2.

### Functional categories and pathway analysis

**Figure 2:**
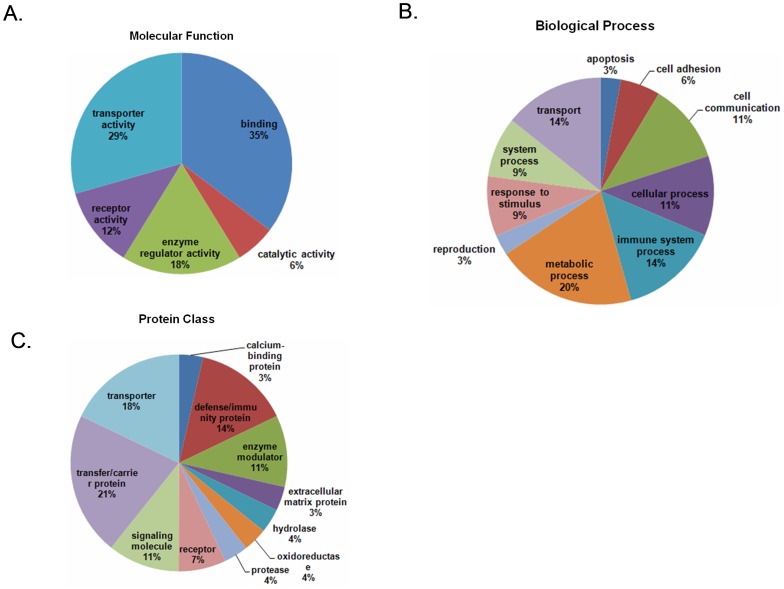
Functional classifications of the identified proteins according to their (A) molecular functions, (B) biological processes and (C) protein class by PANTHER.

To understand the biological effects of severe HFMD on the human serum proteome, the 15 differentially expressed proteins were imported into the PANTHER database [16]. The PANTHER classification system revealed that the proteins could be classified into groups according to their functional properties (Fig. 2A–2C). The most dominant functions of the identified proteins were involved in was binding (35.3%) followed by transporter activity (29.4%) and enzyme regulator activity (17.6%). The biological processes included metabolic process (20.0%), immune system process (14.3%) and transport (14.3%), cellular process (11.4%), cell communication (11.4%). Regarding the protein classes, most of the proteins belonged to transfer/carrier protein (21.4%), transporter (17.9%), defense/immunity protein (14.3%), signaling molecule (10.7%) and enzyme modulator (10.7%).

In the DAVID analysis [17], [18], the Reactome category revealed that 8 proteins were involved in the hemostasis pathway (*p* = 2.22×10^−7^), and 4 proteins were involved in the lipid and lipoprotein metabolism pathways (*p* = 5.82×10^−3^) (Table S2).

### Validation of altered SAA and CLU by ELISA

**Figure 3:**
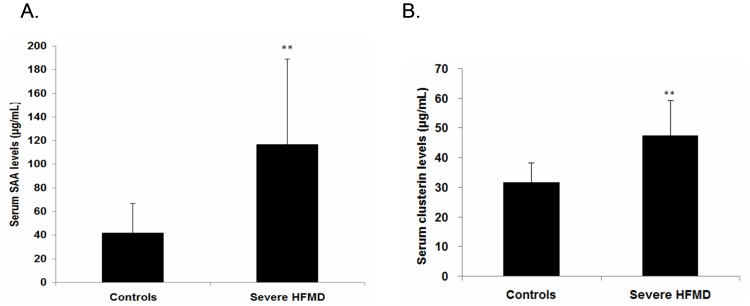
ELISA assay results for the SAA (A) and CLU (B) levels in 20 serum samples from the healthy controls and the severe HFMD groups. The protein levels were expressed as the mean ± the SD. **p<0.01 compared to the healthy controls (independent Student’s *t*-test).

The levels of SAA and clusterin in the sera were quantified by an ELISA assay to confirm the altered expression that was revealed by proteomic analysis and to validate their potentials as biomarkers of severe HFMD. These proteins were chosen because of the commercial availability of antibodies and their greatest activation in 2D-DIGE analysis (SAA, change-fold>+31.19; CLU, change-fold = +6.13). Compared to the controls, the relative levels of SAA and CLU were significantly increased in the severe HFMD cases by 2.77- and 1.82-fold (both *p*<0.01), respectively (Fig. 3A–3B).

## Discussion

Only a small percentage of HFMD cases will further develop into severe HFMD, which often progress very rapidly prior to appropriate treatment. Therefore, the timely and accurate diagnosis of severe HFMD is very important, which can provide the basis for assessing the risk of progression, and planning of appropriate treatment. In the present study, we identified 15 proteins that were differentially expressed in the severe HFMD patients samples compared to the controls with DIGE-based proteomics. A bioinformatics classification of the identified proteins was analyzed by PANTHER [16], and the results are summarized in Fig. 2A–2C. A variety of cellular functions were covered by the identified proteins; these functions included binding, transporter activity and enzyme regulator activity. For biological processes, the identified proteins were involved in many biological processes that included metabolic process, immune system process and transport. Regarding the protein classes, most of the proteins belonged to the transfer/carrier protein, transporter, and defense/immunity protein classes. David analysis [17], [18] revealed that 8 of the identified proteins participated in the hemostasis pathway, and 4 of the proteins were involved in the metabolism of lipids and lipoproteins. Hemostasis is a physiological response that prevents significant bleeding from an injured vessel. In some infectious cases, inflammatory mediators of microbes and/or the host can manipulate the procoagulant/anticoagulant equilibrium and cause severe coagulation disorders [20], [21]. Lipoproteins are complexes of lipids and proteins that are essential for the transport of cholesterol, triglycerides, and fat-soluble vitamins. Disorders that affect lipid and lipoprotein metabolism can lead various diseases [22], [23]. These findings increase our scientific insight and indicate the complexity of the mechanisms of HFMD.

Serum amyloid A (SAA) was found to be the most up-regulated (greater than 30-fold) protein in the severe HFMD patients. SAA is an acute phase reactant that is synthesized by hepatocytes in response to cytokines and other regulatory factors [24]. The physiological response to infections and injuries involve local inflammation and the initiation of events that lead to a wide-ranging systemic response. During the acute-phase response, the hepatic biosynthesis of SAA is up-regulated by pro-inflammatory cytokines, and circulating concentrations of SAA can increase by up to 1000-fold [25]. SAA has been proposed to play important roles in inflammatory reactions: SAA inhibits antibody production in lymphocytes [26], the oxidative burst reaction of neutrophils [27] and chemotaxis of neutrophils and monocytes [28]. In addition, our ELISA assay validated the increased SAA levels in the severe HFMD patients compared to the controls, and this findings is also consistent with the similar proteomics studies in HFMD patients [29]. Our data showed that SAA might be served as a potential biomarker of the clinical diagnosis of severe HFMD.

Clusterin (CLU) was originally identified as an ∼80-kDa major heterodimeric glycoprotein that is secreted from ram rete testis fluid and is predominantly present in most biologic fluids [30], [31]. A number of physiologic functions have been proposed for CLU and include complement regulation, lipid transport, sperm maturation, initiation of apoptosis, endocrine secretion, membrane protection, and promotion of cell interactions [32]. In this study, CLU was found to be the second-most up-regulated protein (more than 6.0-fold) in the severe HFMD patients. A prominent and defined feature of CLU is its induction in neurodegenerative conditions [32]. Strong evidence indicates that the expression of CLU is elevated in a number of pathological conditions that involve injury or chronic inflammation of the brain [33]. CLU contributes to caspase-3-independent brain injury following that the expression levels of CLU were closely related to post-ischemic brain injury [34]. CLU expression has also been reported to increase in affected cortical areas of the brain and to be present in amyloid plaques and in the cerebrospinal fluid [35]–[37]. Since severe HFMD cases can cause neurologic symptoms, including encephalitis, aseptic meningitis, flaccid paralysis and autonomic nervous system dysregulation etc [4], [5]. The increased levels of CLU may constitute the molecular mechanisms of severe HFMD that lead to neurologic complications. The subtle relationship, including the specificity and sensitivity, between the progression of HFMD and altered levels of CLU (and/or SAA) in the serum needs to be further explored and fully assessed.

In the present study, we identified 15 proteins that were differentially expressed between the severe HFMD patients and the normal controls. The identified proteins were classified into different groups according to their molecular functions, biological processes, protein classes and physiological pathways based on bioinformatics analyses. The two most up-regulated proteins, SAA and CLU, were validated as increased in the serum of the severe HFMD samples by ELISA assays, indicating their potentials as the clinical biomarker candidates. In summary, our data showed that the proteomics approach will be of great value for clinical diagnosis, and provided new scientific insights regarding the potential mechanisms of severe HFMD disease.

## Supporting Information

Figure S1 **Example peptide mass spectra used for SAA identification.** The MS/MS spectra of two matched peptides, RGPGGAWAAEVISDAR (A) and FFGHGAEDSLADQAANEWGR (B) for SAA identification.
(TIF)

Figure S2 **Example peptide mass spectra used for CLU identification.** The MS/MS spectra of two matched peptides, EGDDDRTVCR (A) and VTTVASHTSDSDVPSGVTEVVVK (B) for SAA identification.
(TIF)

Table S1 **The detailed characteristic for every individual of both HFMD patients and controls.**
(DOC)

Table S2 **Reactome category of biological pathways associated with identified proteins in David analysis.**
(DOC)
