# Supplementary figures and images for: Serum Amyloid A and Clusterin as Potential Predictive Biomarkers for Severe Hand, Foot and Mouth Disease by 2D-DIGE Proteomics Analysis
Source: PLoS One. 2014 Sep 30;9(9):e108816. doi: 10.1371/journal.pone.0108816 (PMC4182520; doi:10.1371/journal.pone.0108816)

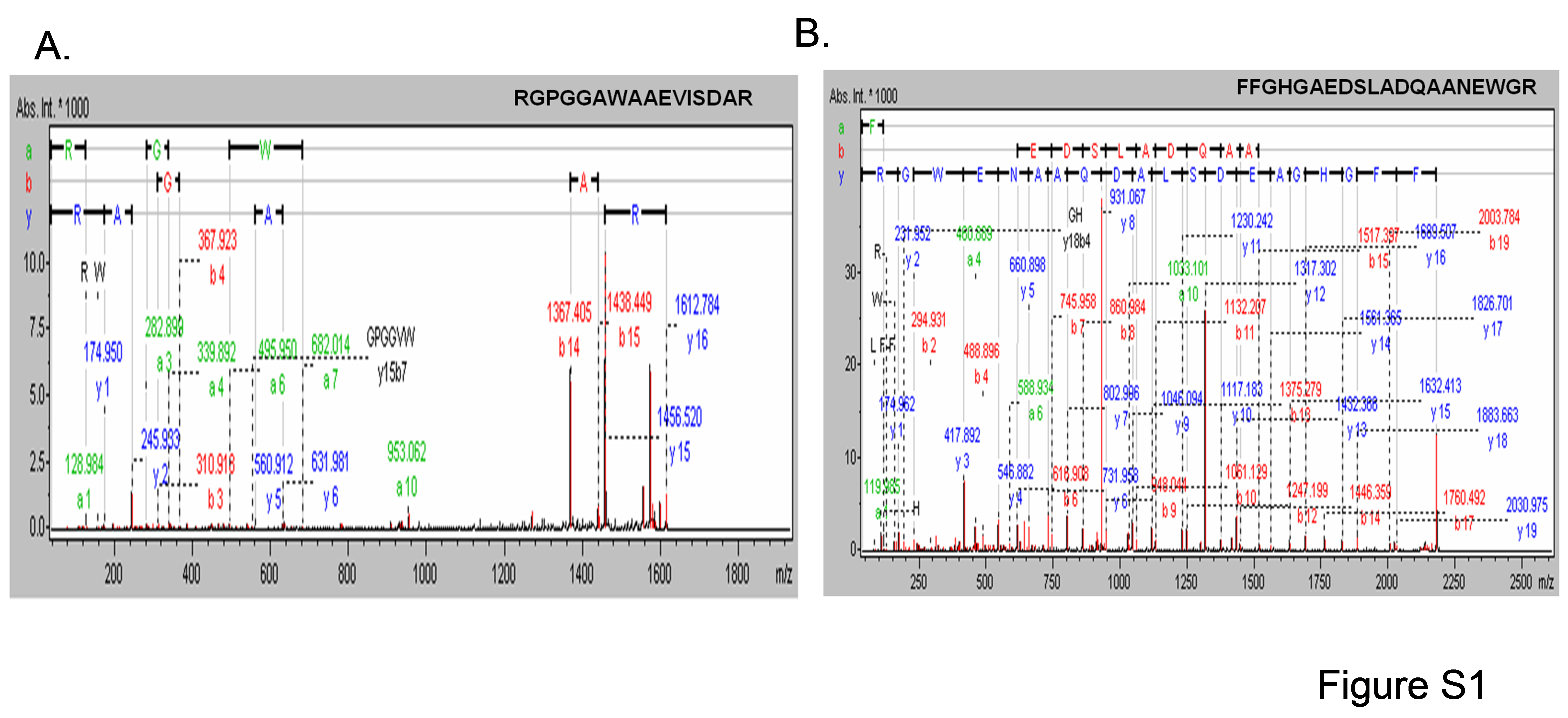

Supplement: Figure S1 — Example peptide mass spectra used for SAA identification. The MS/MS spectra of two matched peptides, RGPGGAWAAEVISDAR (A) and FFGHGAEDSLADQAANEWGR (B) for SAA identification. (TIF) [file pone.0108816.s001.tif]

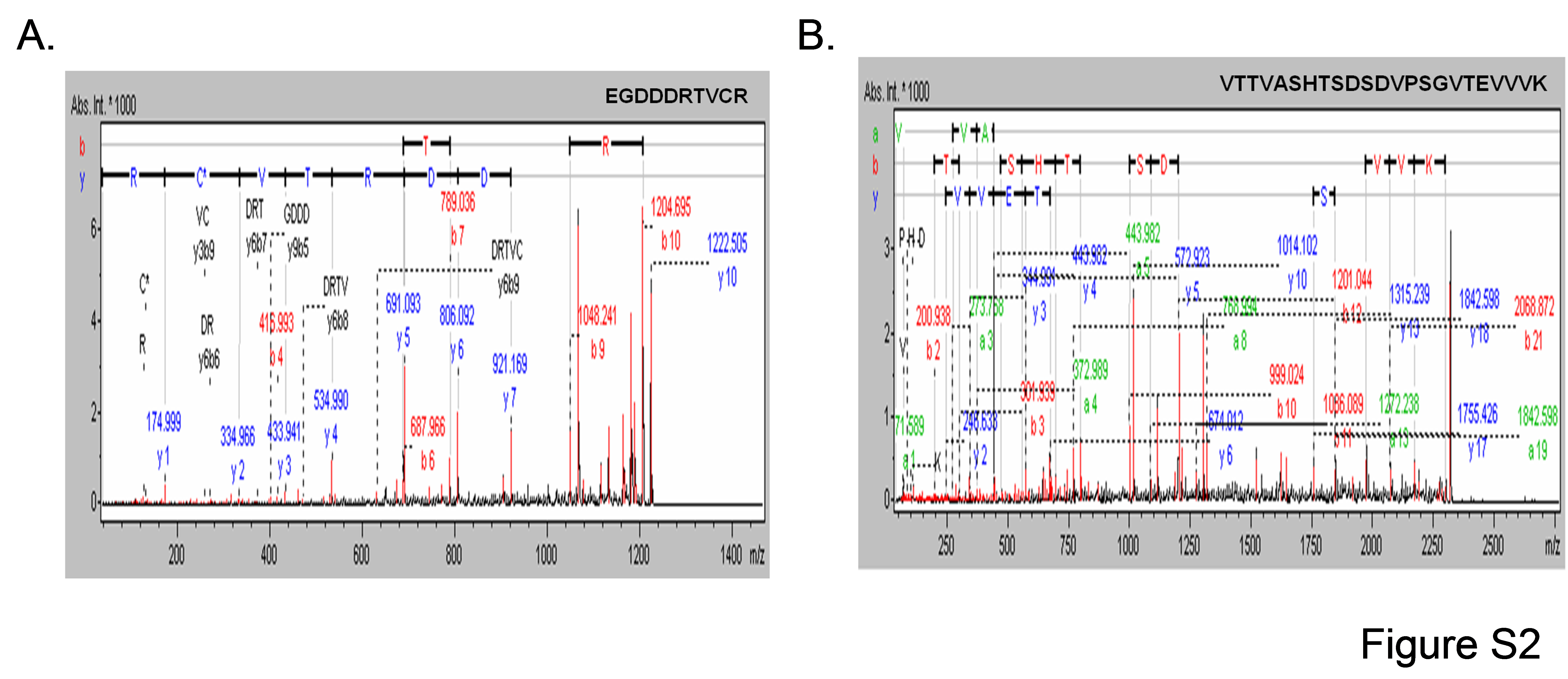

Supplement: Figure S2 — Example peptide mass spectra used for CLU identification. The MS/MS spectra of two matched peptides, EGDDDRTVCR (A) and VTTVASHTSDSDVPSGVTEVVVK (B) for SAA identification. (TIF) [file pone.0108816.s002.tif]
