# Supplementary material for: Serum Amyloid A and Clusterin as Potential Predictive Biomarkers for Severe Hand, Foot and Mouth Disease by 2D-DIGE Proteomics Analysis
Source: PLoS One. 2014 Sep 30;9(9):e108816. doi: 10.1371/journal.pone.0108816 (PMC4182520; doi:10.1371/journal.pone.0108816)
Supplement: Table S1 — The detailed characteristic for every individual of both HFMD patients and controls. (DOC) [file pone.0108816.s003.doc]

Table S1: The detailed characteristic for every individual of both HFMD patients and controls

| Groups | | No | sample ID | gender | age (month/year) |
| --- | --- | --- | --- | --- | --- |
| Severe HFMD | 2D-DIGE and ELISA | 1 | HEI 079 | male | 30/2.50 |
|  |  | 2 | HBI 037 | male | 26/2.17 |
|  |  | 3 | HEI 047 | male | 18/1.50 |
|  |  | 4 | HBI 104 | male | 22/1.83 |
|  |  | 5 | HEI 036 | female | 14/1.17 |
|  |  | 6 | HEI 043 | female | 14/1.17 |
|  |  | 7 | HBI 079 | female | 19/1.58 |
|  |  | 8 | HEI 044 | female | 24/2.00 |
|  | ELISA | 9 | HEI 048 | male | 15/1.25 |
|  |  | 10 | HBI 099 | male | 51/4.25 |
|  |  | 11 | HEI 060 | female | 15/1.25 |
|  |  | 12 | HBI 060 | male | 44/3.67 |
|  |  | 13 | HBI 064 | male | 23/1.92 |
|  |  | 14 | HBI 065 | male | 22/1.83 |
|  |  | 15 | HBI 085 | female | 15/1.25 |
|  |  | 16 | HBI 090 | male | 11/0.92 |
|  |  | 17 | HBI 093 | male | 31/2.58 |
|  |  | 18 | HBI 102 | female | 25/2.08 |
|  |  | 19 | HBI 111 | male | 40/3.33 |
|  |  | 20 | HBI 112 | male | 22/1.83 |
| Normal | 2D-DIEG and ELISA | 1 | HFJ 055 | male | 46/3.83 |
|  |  | 2 | HFJ 017 | male | 36/3.00 |
|  |  | 3 | HFJ 022 | male | 45/3.75 |
|  |  | 4 | HFJ 029 | male | 34/2.83 |
|  |  | 5 | HFJ 028 | female | 45/3.75 |
|  |  | 6 | HFJ 025 | female | 37/3.08 |
|  |  | 7 | HFJ 023 | female | 38/3.17 |
|  |  | 8 | HFJ 006 | female | 34/2.83 |
|  | ELISA | 9 | HFJ 005 | male | 60/5.00 |
|  |  | 10 | HFJ 013 | male | 36/3.00 |
|  |  | 11 | HFJ 021 | male | 32/2.67 |
|  |  | 12 | HFJ 026 | male | 30/2.50 |
|  |  | 13 | HFJ 030 | male | 50/4.17 |
|  |  | 14 | HFJ 031 | male | 41/3.42 |
|  |  | 15 | HFJ 032 | male | 36/3.00 |
|  |  | 16 | HFJ 035 | female | 44/3.67 |
|  |  | 17 | HFJ 034 | female | 46/3.83 |
|  |  | 18 | HFJ 039 | female | 44/3.67 |
|  |  | 19 | HFJ 041 | male | 50/4.17 |
|  |  | 20 | HFJ 044 | male | 58/4.83 |
