# Supplementary material for: Serum Amyloid A and Clusterin as Potential Predictive Biomarkers for Severe Hand, Foot and Mouth Disease by 2D-DIGE Proteomics Analysis
Source: PLoS One. 2014 Sep 30;9(9):e108816. doi: 10.1371/journal.pone.0108816 (PMC4182520; doi:10.1371/journal.pone.0108816)
Supplement: Table S2 — Reactome category of biological pathways associated with identified proteins in David analysis. (DOC) [file pone.0108816.s004.doc]

Table S2 Reactome category of biological pathways associated with identified proteins in David analysis.

| **Entry** | **Pathway** | **Count of identified proteins** | ***P* Value** | **Protein Name** |
| --- | --- | --- | --- | --- |
| 1 | Hemostasis | 8 | 2.22 × 10^-7^ | A2MG_HUMAN,  CLUS_HUMAN, APOA1_HUMAN,  ALBU_HUMAN,  ANT3_HUMAN,  FIBG_HUMAN,  KNG1_HUMAN,  A2AP_HUMAN |
| 2 | Metabolism of lipids and lipoproteins | 4 | 0.00582 | APOC3_HUMAN,  APOA2_HUMAN,  APOA1_HUMAN,  ALBU_HUMAN |
